# Supplementary material for: Innovative Computerized Dystrophin Quantification Method Based on Spectral Confocal Microscopy
Source: Int J Mol Sci. 2023 Mar 28;24(7):6358. doi: 10.3390/ijms24076358 (PMC10094132; doi:10.3390/ijms24076358)
Supplement: Supplementary file 1 [file ijms-24-06358-s001.zip › ijms-2074659-supplementary.pdf]

Supplemantary Table S1.

Clinical, genetic and fluorescence intensity obtained with spectral confocal microscopy data from patients and healthy controls.

PATIENT CODE	DIAGNOSIS	MUSCLE	DIAGNOSTIC CATEGORY (DYSTROPHIN MUTATION)	AGE AT BIOPSY	AGE AT ONSET	DYS 1 MEAN FLUORESCENCE INTENSITY (A.U.)	DYS 2 MEAN FLUORESCENCE INTENSITY (A.U.)	DYS 3 MEAN FLUORESCENCE INTENSITY (A.U.)
SJD_LS_1	DMD	Unknown	Deleted exons 46 -49	6 years	5 years	679,98 ± 20,55	362,17 ± 12,80	287,93 ± 10,69
SJD_LS_2	DMD	Right quadriceps	NM_004006.2):c.6286C>T; p.Gln2096*	6 years	4 years	211,26 ± 7,17	220,50 ± 7,64	259,4± 7,5
SJD_LS_3	DMD	Right quadriceps	NM_004006.2:c.4084C>T; p.Gln1362*	6 years	3 years	433,52 ± 18,71	172,13 ± 5,83	272,77 ± 7,13
SJD_LS_4	BMD	Right quadriceps	DELECION IN FRAME 45>49	6 years	5 years	1550,29 ± 59,28	1665,77 ±75,84	700,06 ±26,28
SJD_LS_5	DMD	Quadriceps	NM_004006.2:c.3578T>A; p.Leu1193*	6 years	18 months	157,11 ± 5,27	257,69 ± 9,34	278,60 ± 8,58
SJD_LS_6	DMD	Quadriceps	NM_004006.2:c.2215G>T; p.Glu739*	5 years	2 years	222,56 ± 8,73	92,14 ± 3,23	74,58 ± 3,02
SJD_LS_7	DMD	Unknown	Deleted exon 53	5 years	2 years	190,40 ± 7,17	93,88 ± 2,93	102,23 ± 3,74
SJD_LS_8	BMD	Unknown	Duplicated exons 6, 7,8 i 9	5 years	4 years	1529,42 ± 53,38	1445,9 ± 52,18	610,84 ± 22,86
SJD_LS_9	DMD	Right quadriceps	NM_004006.2:c.583C>T; p.Arg195*	5 years	2 years	602,03 ± 21,92	494,80 ± 16,47	267,06 ± 6,79
SJD_LS_10	DMD	Right deltoid	Deleted exon 48	4 years	4 years	1800,45 ± 42,09	2518,14 ± 48,20	761,01 ± 28,14
SJD_LS_11	DMD	Unknown	Deleted exons 8-17	4 years	3 years	239,01 ± 9,89	793,68 ± 21,85	330,34 ± 8,02
SJD_LS_12	BMD	Unknown	Deleted exons 13 - 19	4 years	3 years	492,85 ± 14,76	547,3 ± 20,78	267,15 ± 9,64
SJD_LS_13	DMD	Unknown	NM_004006.2:c.6651_6652del; p.Asp2219Phefs*3	4 years	2 years	486,81 ± 14,87	1018,20 ± 34,61	323,31 ± 11,51
CONTROL 1		Trapeze	Right Scapula surgery	15 years		3315,04 ± 63,38	3568,77 ± 41,30	3567,19 ± 52,20
CONTROL 2		Gracilis	Gracilis muscle transfer	5 years		3756,77 ± 29,98	2689,88 ± 82,27	3704,91 ± 36,88
CONTROL 3		Flexor	Compression of ulnar nerve	18 years		1214,11 ± 32,08	1435,76 ± 42,15	1214,11 ± 32,05
CONTROL 4		Pectoral	Cardiomyopathy	9 years		3755,71 ± 35,13	2767,77 ± 88,78	3456,18 ± 63,63
CONTROL 5		Femoral bíceps	Osteochondroma	14 years		2567,42 ± 48,13	2909,11 ± 5709	2673,72 ± 63,62
CONTROL 6		Biceps	Derotatory osteotomy of humerus	14 years		2641,25 ± 53,47	2544,78 ± 70,20	2642,73 ± 76,45
